# Murine Pancreatic Adenocarcinoma Dampens SHIP-1 Expression and Alters MDSC Homeostasis and Function

**DOI:** 10.1371/journal.pone.0027729

**Published:** 2011-11-22

**Authors:** Shari Pilon-Thomas, Nadine Nelson, Nasreen Vohra, Maya Jerald, Laura Pendleton, Karoly Szekeres, Tomar Ghansah

**Affiliations:** 1 Department of Molecular Medicine, College of Medicine, University of South Florida, Tampa, Florida, United States of America; 2 Immunology Program, H. Lee Moffitt Comprehensive Cancer Center and Research Institute, Tampa, Florida, United States of America; Penn State Hershey Cancer Institute, United States of America

## Abstract

**Background:**

Pancreatic cancer is one of the most aggressive cancers, with tumor-induced myeloid-derived suppressor cells (MDSC) contributing to its pathogenesis and ineffective therapies. In response to cytokine/chemokine receptor activation, src homology 2 domain-containing inositol 5′-phosphatase-1 (SHIP-1) influences phosphatidylinositol-3-kinase (PI3K) signaling events, which regulate immunohomeostasis. We hypothesize that factors from murine pancreatic cancer cells cause the down-regulation of SHIP-1 expression, which may potentially contribute to MDSC expansion, and the suppression of CD8^+^ T cell immune responses. Therefore, we sought to determine the role of SHIP-1 in solid tumor progression, such as murine pancreatic cancer.

**Methodology and Principal Findings:**

Immunocompetent C57BL/6 mice were inoculated with either murine Panc02 cells (tumor-bearing [TB] mice) or Phosphate Buffer Saline (PBS) (control mice). Cytometric Bead Array (CBA) analysis of supernatants of cultured Panc02 detected pro-inflammatory cytokines such as IL-6, IL-10 and MCP-1. TB mice showed a significant increase in serum levels of pro-inflammatory factors IL-6 and MCP-1 measured by CBA. qRT-PCR and Western blot analyses revealed the *in vivo* down-regulation of SHIP-1 expression in splenocytes from TB mice. Western blot analyses also detected reduced SHIP-1 activity, increased AKT-1 and BAD hyper-phosphorylation and up-regulation of BCL-2 expression in splenocytes from TB mice. *In vitro*, qRT-PCR and Western blot analyses detected reduced SHIP-1 mRNA and protein expression in control splenocytes co-cultured with Panc02 cells. Flow cytometry results showed significant expansion of MDSC in peripheral blood and splenocytes from TB mice. AutoMACS sorted TB MDSC exhibited hyper-phosphorylation of AKT-1 and over-expression of BCL-2 detected by western blot analysis. TB MDSC significantly suppressed antigen-specific CD8^+^ T cell immune responses *in vitro*.

**Conclusion/Significance:**

SHIP-1 may regulate immune development that impacts MDSC expansion and function, contributing to pancreatic tumor progression. Thus, SHIP-1 can be a potential therapeutic target to help restore immunohomeostasis and improve therapeutic responses in patients with pancreatic cancer.

## Introduction

Pancreatic cancer is a fatal malignancy, ranking as the fourth most common cause of cancer-related mortalities in the United States [1]. Pancreatic cancer prognoses are poor due to its late detection, leading to advanced stages and inoperability at presentation, in most patients [1]. In addition, pancreatic cancer is resistant to a majority of chemotherapies, radiation and immunotherapies [2]. The standard chemotherapeutic drug for pancreatic cancer treatment is Gemcitabine [2]. Gemcitabine prolongs survival, provides short-term symptomatic improvement in only a few patients and rarely leads to cures [2]. Pancreatic tumor-bearing hosts release soluble inflammatory factors that lead to the expansion of immunosuppressive myeloid-derived suppressor cells (MDSC) [3]. MDSC suppress CD8^+^ T cell responses thus curtailing the efficacy of a plethora of cancer therapies [4]. Therefore, it is imperative to understand the molecular signaling events that regulate MDSC homeostasis and function in a pancreatic tumor microenvironment. The identification of new molecular targets is critical to improve anti-tumor immune responses against pancreatic cancer.

Src homology 2 domain-containing inositol 5′ -phosphatase (SHIP-1), a 145-kDa protein, is a critical regulator of numerous biological processes in hematopoietic cells such as proliferation, differentiation, activation, migration, and survival/apoptosis, via its ability to regulate the phosphatidylinositol-3-kinase (PI3K) pathway [5]. In response to cytokine or growth factor signaling, the PI3K phospholipid product PIP_3_ recruits pleckstrin homology (PH) domain containing kinases such as protein kinase B (PKB also known as AKT) [6]. AKT regulates apoptosis/survival signaling events that influence the outcome of immune responses [6]. SHIP-1, or SHIP-2 (an isoform expressed in non-hematopoietic and hematopoietic cells [7]) can de-activate the PI3K pro-survival signaling pathways by hydrolyzing PI-3,4,5-P_3_ phospholipid product to yield PI-3,4-P_2_, thus maintaining immune cell homeostasis [5]. SHIP-1 has recently been implicated as a possible tumor suppressor in hematopoietic cancers [5] as its protein expression is notably reduced or mutated in many leukemias and lymphomas [8], [9], [10], [11]. However, SHIP-1's role in solid tumor progression has not been fully investigated. SHIP-2 has not been identified as a tumor suppressor but there is preliminary evidence that suggests it may promote tumor progression [12], [13]. Thus, the regulation of PI3K pathways is important to maintain immune homeostasis and prevent tumorigenesis [5].

SHIP deficient mice have perturbed cytokine and chemokine production resulting in a pro-inflammatory phenotype [14]. Myeloid progenitors of SHIP deficient mice are highly responsive to modulation with cytokine, growth factors or chemokines in comparison to their wild-type counterparts [14], [15]. As a result, SHIP deficient mice develop splenomegaly, due to increased myelopoiesis [14]. They also develop a myeloproliferative disease (MPD) with some resemblance to chronic myeloid leukemia (CML) in humans [14]. Ghansah et al reported that SHIP deficient mice have a significant expansion of immunosuppressive Gr1^+^CD11b^+^ MDSC, which prevented allograft rejection by suppressing allogeneic T cell responses *in vivo* and *in vitro* [16], [17]. MDSC are a heterogeneous population of immature myeloid cells that expand due to changes in the microenvironment in response to inflammation/cancer, ultimately suppressing T cell responses by a variety of mechanisms [18]. A significant expansion of immunosuppressive regulatory T cells (Treg), which can also lead to suppression of T cell immune responses, is observed in SHIP deficient mice [19]. SHIP deficient mice are also noted to have an expansion of macrophages that secrete high levels of IL-6 in response to increasing IgG production [20]. This in turn leads to reduced B cell percentages and enhanced myeloid-cell development in these mice [21]. SHIP deficient mice are potentially more susceptible to the facilitation of solid tumor growth [5] which is likely due to the loss of immune cell homeostasis.

In this study, we evaluated the role of SHIP-1 in the development of non-hematopoietic cancer (solid pancreatic tumor) and its effects on immunosuppressive leukocytes. We hypothesized that murine pancreatic cancer creates an inflammatory environment, which alters the expression of SHIP-1 in leukocytes. Furthermore, this altered SHIP-1 expression negatively affects MDSC homeostasis and function, thus suppressing anti-tumor immune responses. Therefore, we propose that SHIP-1 may act as a tumor suppressor and a therapeutic target for the treatment of non-hematopoietic solid tumors such as pancreatic cancer.

## Results

### Murine Panc02 cells modulate pro-inflammatory factors *in vivo*

**Figure 1:**
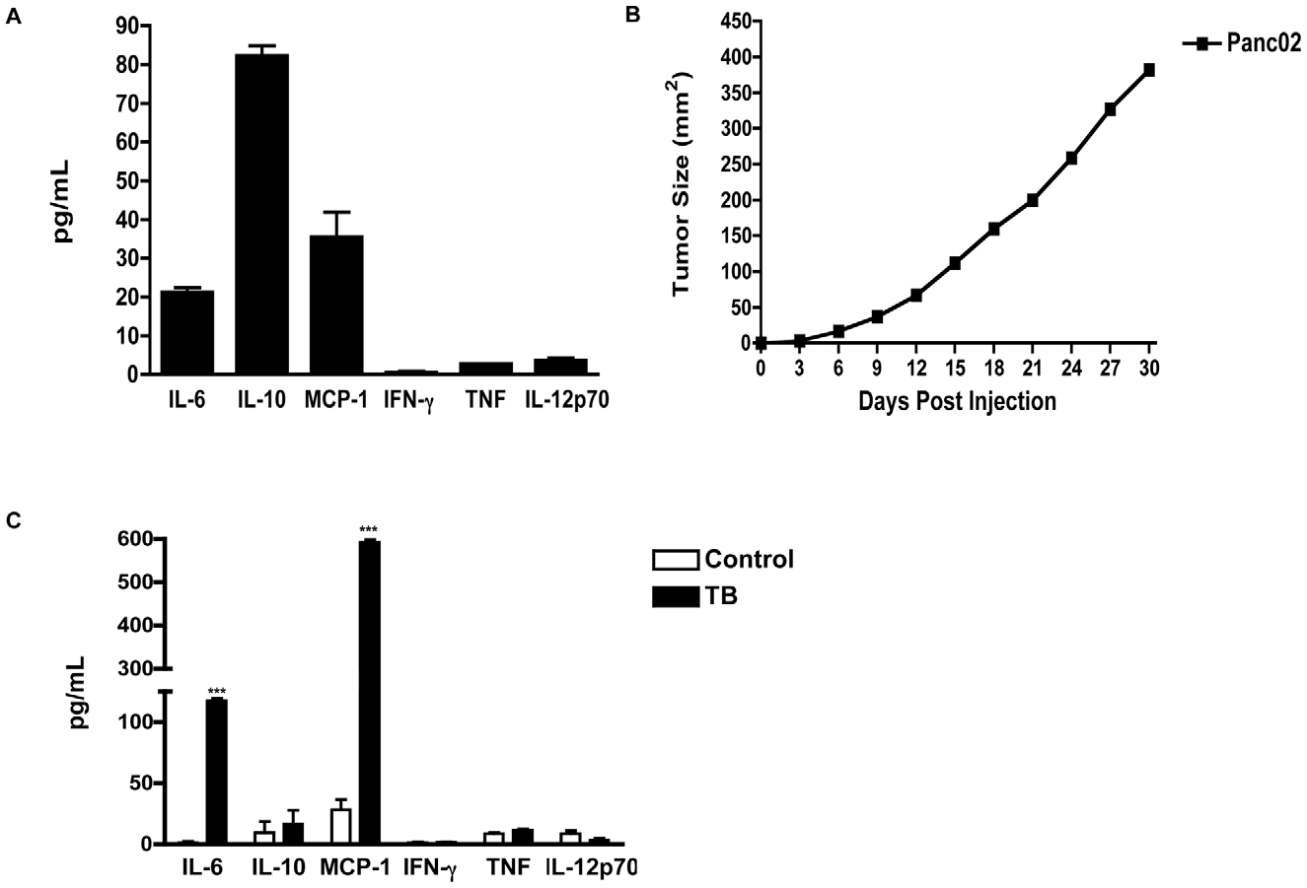
Increase in Pro-inflammatory factors in murine Panc02 cells and TB mice. (**A**) Pro-inflammatory cytokine production by Panc02 cells as determined by CBA and flow cytometry analysis of culture supernatant. (**B**) Growth curve of TB mice inoculated with murine Panc02 cells. (**C**) Cytokine profiles of TB and control mice as measured by CBA assay. Represented is the mean ± S.E.M. of control (n = 4) compared to TB (n = 4) mice. ***p<0.001 (by two-tailed Student's *t* test).

**Figure 2:**
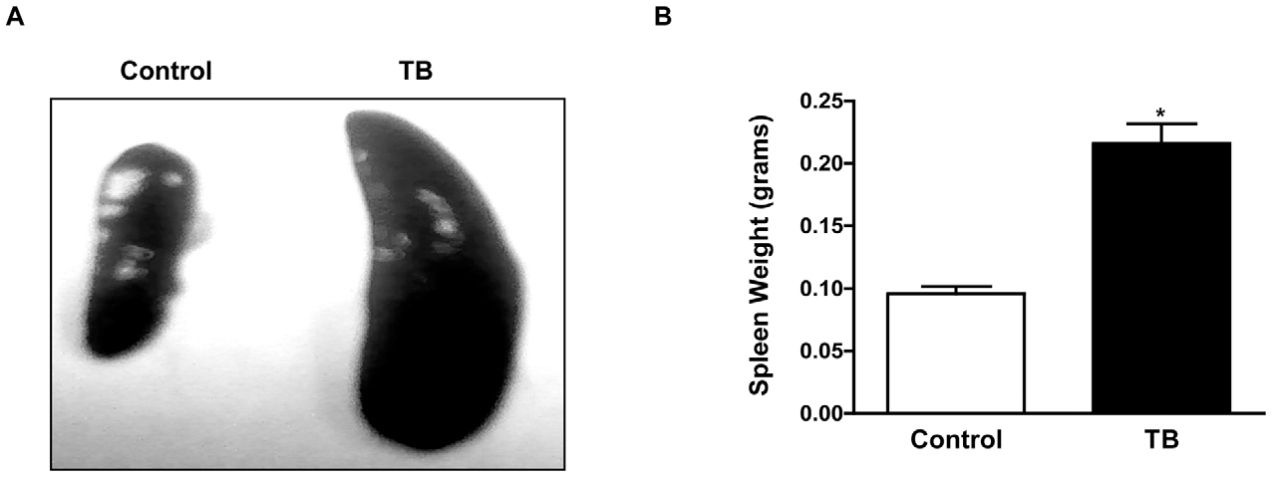
Splenomegaly in TB mice. (**A**) Spleens from TB and control. mice. (**B**) Weights of spleens from TB and control mice. Represented is the mean ± S.E.M. of control (n = 4) compared to TB (n = 4) mice. ***p<0.001; *p<0.05 (by two-tailed Student's *t* test).

Tumor-derived soluble factors, such as cytokines and chemokines, in the tumor microenvironment can modulate leukocyte development and influence immune responses [22]. Therefore, we wanted to evaluate the pro-inflammatory factors produced by murine Panc02 cells *in vitro*. Results from the Inflammatory Cytometric Bead Analysis (CBA) Kit detected pro-inflammatory factors Interleukin-6 (IL-6), Interleukin-10 (IL-10) and Monocyte Chemoattractant Protein-1 (MCP-1) to a greater extent than Tumor Necrosis Factor (TNF), Interferon gamma (IFN-γ and Interleukin-12p 70 (IL-12p70) in the supernatants of cultured murine Panc02 cells (**Figure 1a**). Next, 1.5×10^5^ murine Panc02 cells were subcutaneously inoculated into C57BL/6 mice (tumor-bearing mice (TB)). Control mice received Phosphate Buffer Saline (PBS). Post tumor inoculation, we evaluated tumor progression and weight of TB mice for approximately 30 days. An exponential increase in tumor growth was observed in TB mice (**Figure 1b**) but no significant difference was seen in weight of TB compared to control mice (*data not shown*). We observed that TB mice usually succumbed to death within 25–30 days post Panc02 inoculation, depending on tumor load. Serum was evaluated to determine whether there were changes in the production of pro-inflammatory factors in TB mice. CBA analysis revealed a significant increase in the production of pro-inflammatory factors IL-6 and MCP-1 but no significant change in the production of IL-10, TNF, IFN-γ or IL-12p70 in serum collected from TB compared to control mice (**Figure 1c**). Spleens from TB mice exhibited splenomegaly and weighed significantly more compared to those from control mice (**Figure 2a and 2b**). It is important to note that spleens from TB mice have a similar phenotype to spleens from SHIP deficient mice with significant splenomegaly observed in both [14].

### TB splenocytes have a reduction in SHIP-1 expression

**Figure 3:**
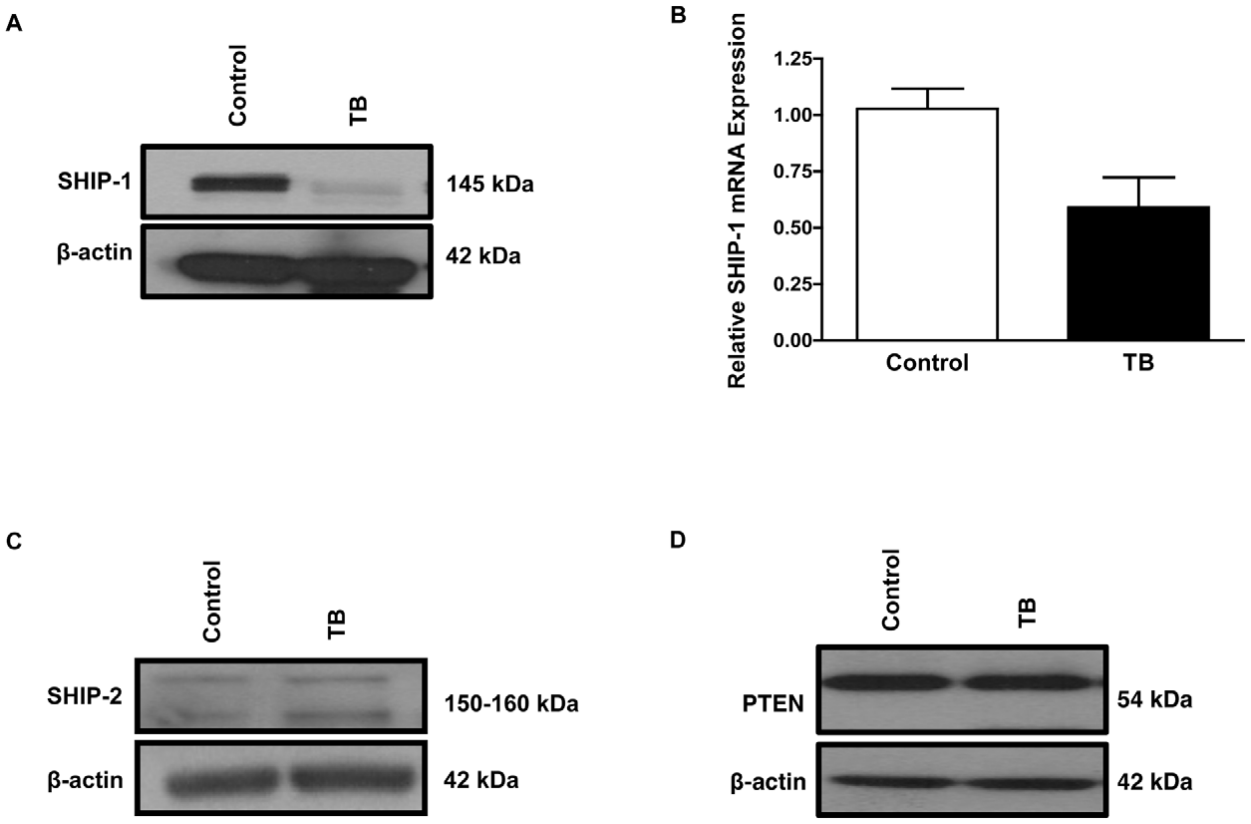
Down-regulation of SHIP-1 in splenocytes from TB mice. (**A**) Western blot analysis of SHIP-1 protein expression in whole splenocytes from control and TB mice. *(**B**) qRT-PCR analysis of SHIP-1 mRNA expression in whole splenocytes from control and TB mice. (**C**) Western blot analysis of SHIP-2 protein expression in whole splenocytes from control and TB mice. (**D**) PTEN protein expression in whole splenocytes from control and TB mice. To control for equal protein loading the blot was re-probed with an antibody specific for β-actin. *Represented is the mean ± S.E.M. of control (n = 3) compared to TB (n = 3) mice. *p*<0.05 (by two-tailed Student's *t* test).

SHIP expression is sensitive to cytokine/chemokine responses and immune stress [23]. Therefore, we hypothesized that SHIP-1 expression would be altered in our pancreatic TB mice due to an increase in the pro-inflammatory factors we observed in these mice. Western blot analyses revealed reduced SHIP-1 protein expression in the splenocytes from TB compared to control mice (**Figure 3a**). This SHIP-1 antibody recognizes the carboxy terminus of mouse origin. Next, we wanted to investigate whether the reduction in SHIP-1 protein expression in TB splenocytes was due to a transcriptional or post-translational event. Quantitative Reverse Transcription Polymerase Chain Reaction (qRT-PCR) results detected a decrease in SHIP-1 mRNA expression in TB compared to control splenocytes (**Figure 3b**). We then evaluated SHIP-2 and PTEN protein expression, which are both critical inositol phosphatases that hydrolyze the PI3K phospholipid product PI-3,4,5-P_3_ [5]. Western blot results revealed no significant difference in the protein expression of SHIP-2 or PTEN in splenocytes from TB compared to control mice (**Figure 3c and 3d**). Thus far, our data suggests that a pancreatic tumor-induced inflammatory microenvironment alters SHIP-1 protein expression and may potentially affect downstream survival/apoptotic-signaling pathways in immune cells.

### TB splenocytes have reduced SHIP-1 activity

**Figure 4:**
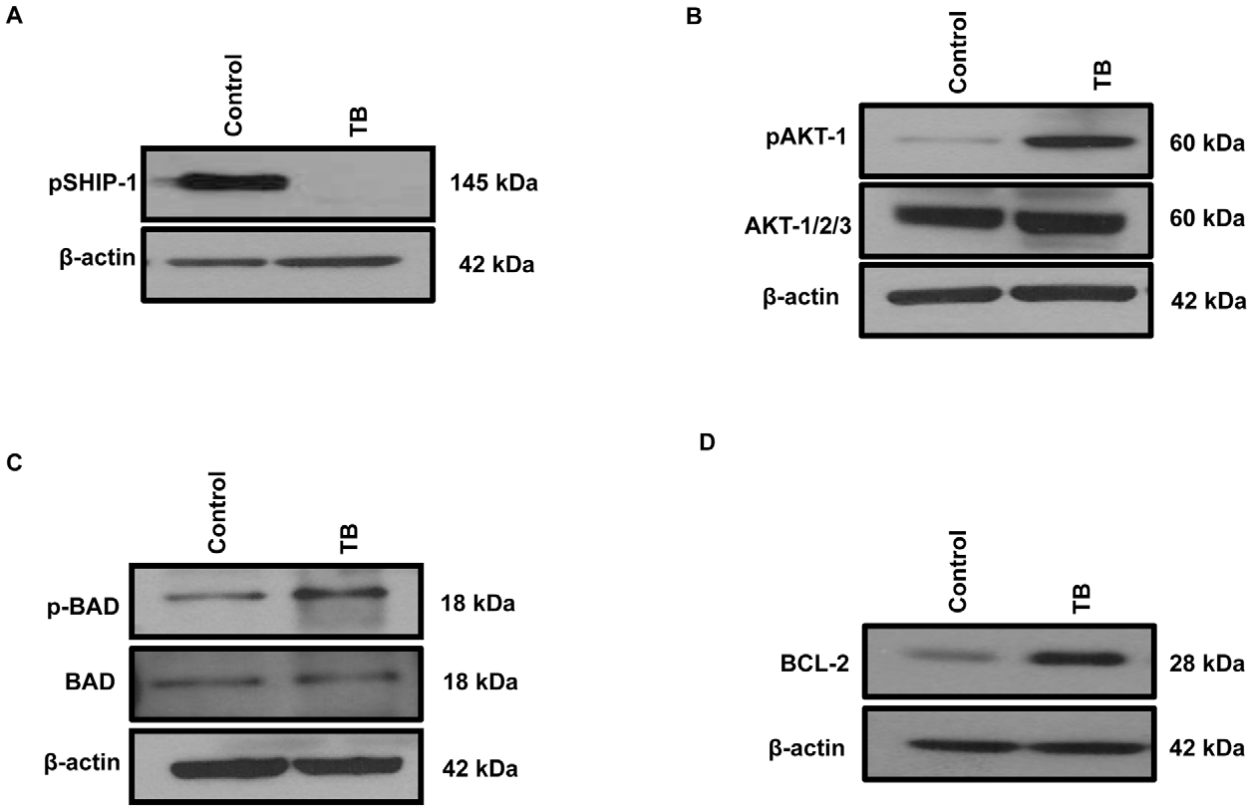
Activation of pro-survival pathways in splenocytes TB mice. (**A**) Western blot analysis of phospho-SHIP-1 (Tyr1020) expression in whole splenocytes from control and TB mice. (**B**) Western blot analysis of phospho-AKT-1 (Ser473) and AKT 1/2/3 protein expression in whole splenocytes from control and TB mice. (**C**) Western blot analysis of phospho-BAD (Ser112) and BAD protein expression in whole splenocytes from control and TB mice, (**D**) Western blot analysis of BCL-2 protein expression in whole splenocytes from control and TB mice. To control for equal protein loading the blot was re-probed with an antibody specific for β-actin. Control (n = 3) compared to TB (n = 3) mice.

Phosphorylation of SHIP-1 on tyrosine residues (917 and 1020) causes it to associate with the adapter protein Shc and translocate to the plasma membrane, which is crucial for its enzymatic activity [24], [25], [26]. We therefore evaluated the phosphorylation status of SHIP-1 in our TB mice. Western blot analyses did not detect phosphorylation of tyrosine 1020 on SHIP-1, in splenocytes from TB compared to control mice (**Figure 4a**). This particular phospho-SHIP 1020 antibody recognizes endogenous level of SHIP-1 in human, mouse and rat. We were unable to evaluate tyrosine phosphorylation of residue 917 on SHIP-1 proteins expressed in lysates from TB and control mice, as there are no commercially available antibodies.

### TB splenocytes have increased AKT activity

SHIP ablation, reduction in its expression or loss of its function due to mutations, leads to constitutively activated AKT/PKB and its downstream signaling events that regulate cell survival [5]. Therefore, we examined whether AKT/PKB, a downstream target of SHIP-1, was hyper-phosphorylated in splenocytes obtained from TB mice compared to control. Western Blot results revealed hyper-phosphorylation of AKT-1 at Ser473 in whole splenocytes from TB compared to control mice (**Figure 4b**). Total AKT levels were detected by using an AKT1/2/3 specific antibody, which showed no difference in AKT protein expression between TB and control splenocytes. It is important to note that phosphorylation at Thr308 and Ser473 are both required for full activation of AKT/PKB [27]. However, western blot analyses did not detect phosphorylation of AKT-1 at Thr308 in TB compared to control mice (*data not shown*). Therefore, phosphorylation of AKT-1 at Ser473 alone may alter homeostasis and function of immune cells in this murine pancreatic tumor model.

### TB splenocytes have increased BCL-2 expression

Next, we wanted to examine the phosphorylation and expression of B-cell lymphoma 2 Associated Death promoter (BAD), a key pro-apoptotic protein upstream of B-cell lymphoma 2 (BCL-2) and regulated by AKT activity [28]. Western Blot results showed hyper-phosphorylation of BAD at Ser112 in splenocytes from TB mice compared to control mice (**Figure 4c**). However, total protein expression of BAD did not change (**Figure 4c**). Phosphorylated BAD is sequestered in the cytosol, which causes the release of anti-apoptotic protein, BCL-2 [28]. It has been reported that the over-expression of BCL-2 in many cancers leads to the loss of leukocyte homeostasis and their resistance to normal apoptotic processes [29], [30]. Therefore, we examined whether there was a difference in the expression of BCL-2 in splenocytes from TB and control mice. Western blot results revealed an up-regulation in BCL-2 expression in splenocytes from TB mice (**Figure 4d**) compared to control. Thus far, our western blot data suggests that survival pathways may be activated in leukocytes from TB compared to control mice likely as a result of suppression of SHIP-1 expression by the pancreatic tumor microenvironment.

### Murine Panc02 cells down-regulate SHIP-1 expression *in vitro*

**Figure 5:**
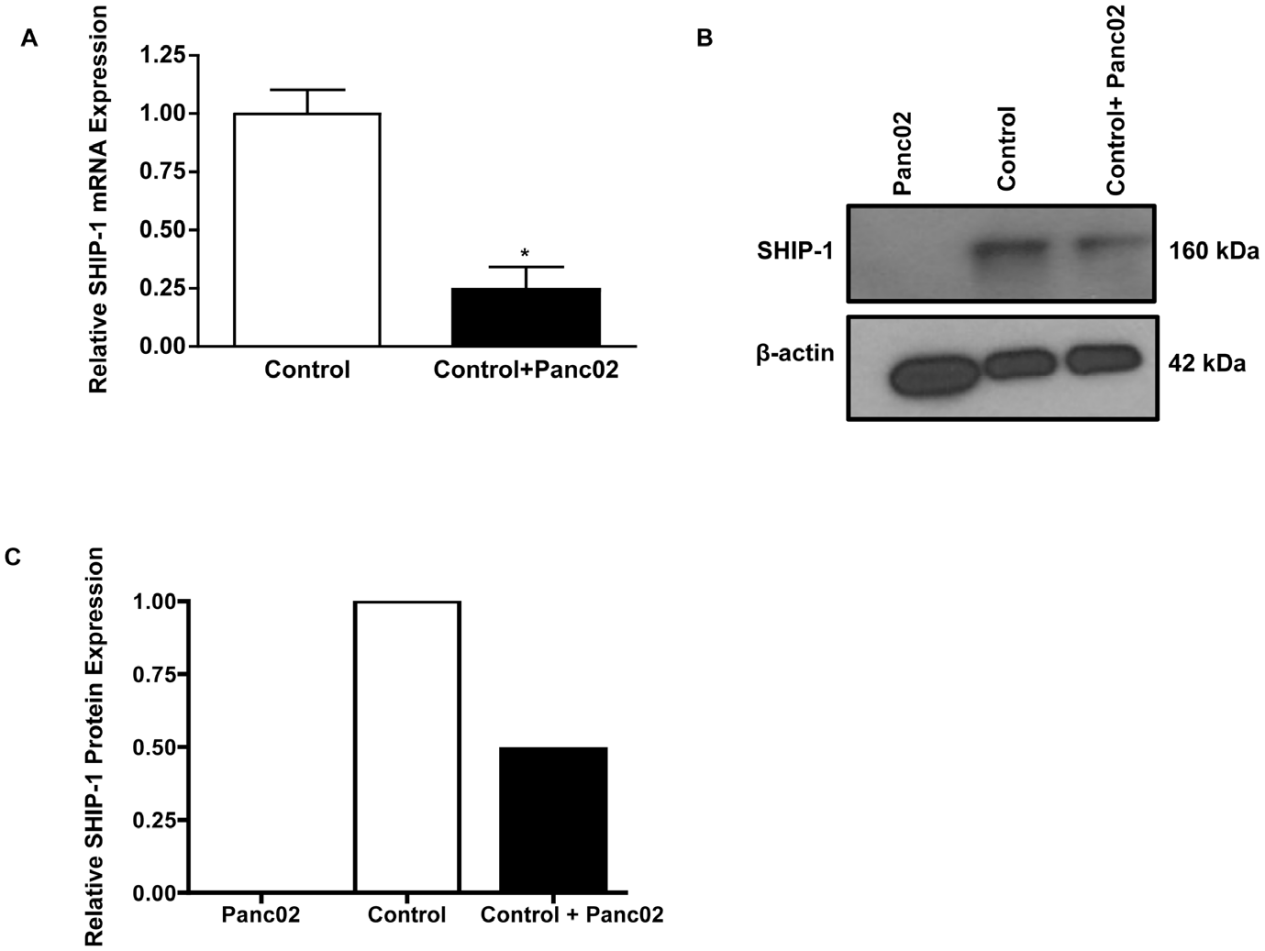
Down-regulation of SHIP-1 in control splenocytes co-cultured with Panc02 cells. *(**A**) qRT-PCR analysis of SHIP-1 mRNA expression in control splenocytes alone and control splenocytes co-cultured with Panc02 cells *in vitro*. (**B**) Western blot analysis of SHIP-1 protein expression in Panc02 cells, control splenocytes and control splenocytes co-cultured with Panc02 cells *in vitro*. To control for equal protein loading the blot was re-probed with an antibody specific for β-actin. (**C**) Representative densitometric quantification of western blot data in panel B. *Represented is the mean ± S.E.M. of three independent experiments. p<0.05 (by two-tailed Student's t test).

Hematopoietic cell proliferation and survival is regulated by SHIP-1 mRNA and protein expression which is cytokine-dependent [31], [32], [33], [34]. Thus far, our *in vivo* data shows that murine Panc02 inoculation into immunocompetent mice suppresses SHIP-1 expression, thereby potentially negatively affecting leukocyte homeostasis. However, to determine whether Panc02 tumor cells down-regulate SHIP-1 expression, outside of the context of *in vivo* cellular interactions, we performed an *in vitro* assay with murine Panc02 cells and control splenocytes. qRT-PCR analysis revealed a significant decrease in SHIP-1 mRNA expression in control splenocytes co-cultured with Panc02 cells compared to control splenocytes cultured alone (**Figure 5a**). In addition, western blot results revealed greater than a a 2-fold reduction in SHIP-1 protein expression in control splenocytes co-cultured with Panc02 cells compared to control splenocytes cultured alone (**Figure 5b**). Western blot results also showed that Panc02 cells do not express SHIP-1 (**Figure 5b**). Representative western blot data of SHIP-1 expression in **Figure 5b** was quantified using densitometry analysis (**Figure 5c**). These results correlate with our *in vivo* data and show that Panc02 cells are able to suppress SHIP-1 mRNA and protein expression *in vitro*.

### TB splenocytes have loss of homeostasis of MDSC

**Figure 6:**
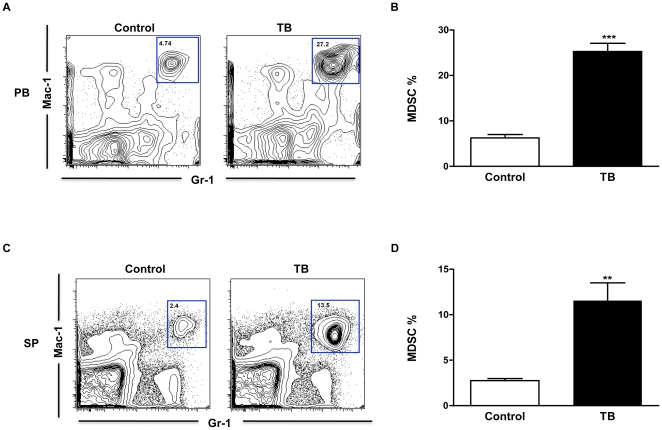
Expansion of MDSC in peripheral blood and splenocytes from TB mice. (**A**) MDSC percentages in peripheral blood (PB) from TB and control mice. Shown are representative flow cytometry results of Mac-1^+^Gr-1^+^ MDSC. (**B**) Represented is the Mean ± S.E.M of the relative percentages of MDSC in TB and control mice. (**C**) MDSC percentages in splenocytes (SP) from TB and control mice. Shown are representative flow cytometry results of Mac-1^+^Gr-1^+^ MDSC. (**D**) Represented is the Mean ± S.E.M of the relative percentages of MDSC in the splenocytes of TB and control mice. Control (n = 3) compared to TB (n = 3) mice **p<0.01; ***p<0.001 (by two-tailed Student's *t* test).

MDSC activation and accumulation are driven by multiple factors associated with pro-inflammatory cytokines produced mainly by tumor cells [35]. In addition, MDSC and myeloid cells are expanded in SHIP deficient mice and are resistant to apoptosis possibly due to the constitutive activation of AKT/PKB [15]. Since our western blot data showed down-regulation of SHIP-1 protein expression and activation of several pro-survival proteins in splenocytes from TB mice, we wanted to determine if there was an expansion of MDSC in these mice. Flow cytometry results revealed a significant expansion of Gr-1^+^CD11b^+^ MDSC in peripheral blood (PB) (**Figure 6a and 6b**) and splenocytes (SP) (**Figure 6c and 6d**) of TB compared to control mice. These results suggest that murine pancreatic cancer causes the down-regulation of SHIP-1 expression which, directly or indirectly, negatively affects immune cell development and may lead to exacerbated suppression of anti-tumor immunity in pancreatic TB mice.

### TB MDSC suppress Ag-specific T cell responses *in vitro*

**Figure 7:**
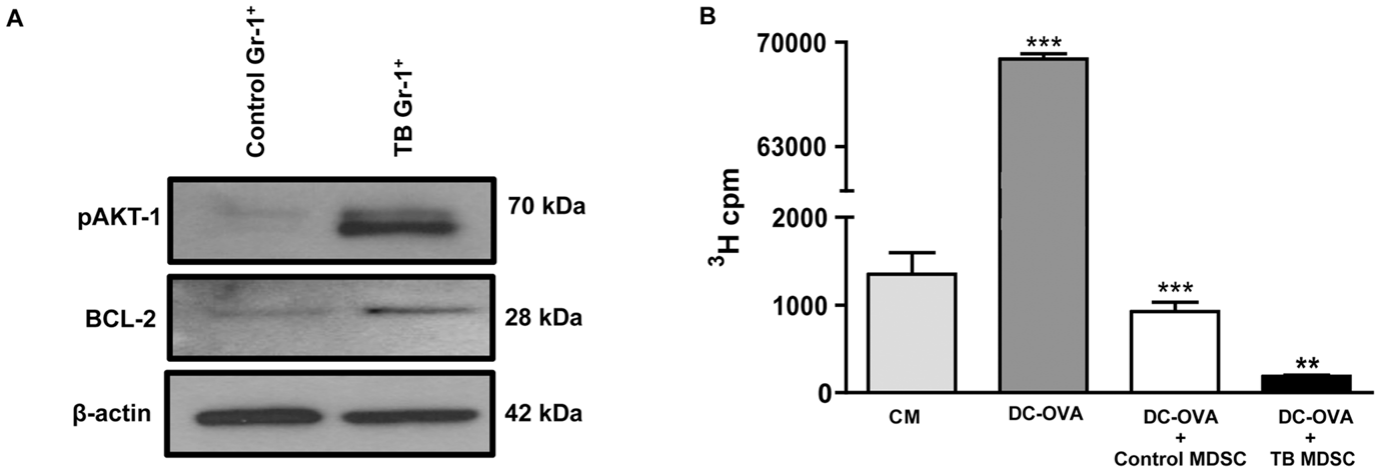
Loss of homeostasis and function of MDSC in splenocytes from TB mice. (**A**) Western blot analysis of phospho-AKT-1 (Ser473) and BCL-2 protein expression in Gr-1^+^ enriched MDSC splenocytes from TB and control mice. To control for equal protein loading the blot was re-probed with an antibody specific for β-actin. (**B**) TB Gr1^+^ MDSC significantly suppress antigen-specific T cell responses (OT-I specific (CD8^+^) T cells primed with OVA-peptide pulsed DC (DC-OVA)) compared to control Gr1^+^ MDSC *in vitro*. Controls for the assay included T cells incubated alone in complete medium (CM) or DC-OVA. Control (n = 3) compared to TB (n = 3) mice. **p<0.01; ***p<0.001 (by two-tailed Student's *t* test).

SHIP deficiency leads to an expansion of MDSC and a suppression of T cell immune responses in mice [16], [17]. As previously shown, flow cytometry results revealed a significant expansion of MDSC in TB splenocytes (**Figure 6c and 6d**). Next we purified Gr-1^+^ cells (MDSC) from TB and control splenocytes using autoMACS enrichment and made protein lysates of these Gr-1^+^ cells. Western blot data showed that Gr1^+^ cells (MDSC) from splenocytes from TB mice had hyper-phosphorylated AKT-1 and over expressed BCL-2 compared to Gr1^+^ cells from control mice (**Figure 7a**). Since our western blot data firmly suggests a loss of homeostasis due to an increase in AKT-1 activity and up-regulation of BCL-2 expression, we then wanted to examine if there are any functional differences in TB MDSC (Gr1^+^ cells) compared to control. To examine suppressor activity, Gr1^+^ cells were purified from splenocytes of control and TB mice as previously described and added to a culture of OT-I cells (OVA_SIINFEKL_-specific CD8^+^ T cells) stimulated with dendritic cells (DC) pulsed with OVA_SIINFEKL_ peptide. Proliferation was measured by ^3^H-thymidine uptake. As shown in **Figure 7b**, both control and TB MDSC (Gr1^+^ cells) were capable of suppressing T cell proliferation. However, a significant increase in suppressor function was measured in TB MDSC compared to control MDSC. These results strongly illustrate that TB MDSC (Gr1^+^ cells) exhibit increased suppression of antigen-specific CD8^+^ T cell immune responses on a cell-to-cell basis in comparison to control MDSC. This implies that MDSC from pancreatic tumor microenvironment may be functionally suppressive and may contribute to the aggressive nature of pancreatic cancer.

## Discussion

SHIP-1, as a negative regulator of PI3K signaling in hematopoietic cells, plays a critical role in hematological malignancies [5]. SHIP-1 has recently been proposed to function as a tumor suppressor in leukemias and lymphomas [8], [9], [10], [11]. However, the role of SHIP-1 in solid malignancies has not been fully investigated. In this study, we demonstrated that SHIP-1 may play a role in the progression of pancreatic tumors by altering the homeostasis and function of immunosuppressive MDSC and potentially other immune cells. Our studies suggest that SHIP-1 may act as a tumor suppressor and may be a novel therapeutic target for the reduction of tumor-induced immune suppression.

Panc02 cells secrete a number of inflammatory factors *in vitro*. Inoculation of mice with Panc02 cells led to an inflammatory phenotype *in vivo* and significant splenomegaly in Panc02 TB mice. Differences in cytokine production of *in vitro* cultured Panc02 cells compared to *in vivo* TB serum can be attributed to the vast array of tumor-cell interactions that occur *in vivo*. Reduced expression of SHIP-1 in TB splenocytes may be the result of the increased inflammatory cytokines detected in their serum. Results from co-culture of control splenocytes with Panc02 cells show that Panc02 factors (soluble and non-soluble) down-regulate SHIP-1 mRNA and protein expression *in vitro*. Recently, research findings have identified a number of transcription factors and particular microRNAs as regulators of SHIP-1 gene expression [10], [36], [37], [38]. These and other potential cytokine-dependent transcriptional regulators of SHIP-1 expression and lymphocyte development are currently being investigated.

The PI3K/AKT pathway is constitutively activated in some pancreatic cancers [39]. SHIP-1 negatively regulates PI3K/AKT pathways, which control apoptosis/survival in hematopoietic cells [40]. In this study, not only did we observe suppressed SHIP-1 expression in this pancreatic tumor microenvironment but also reduced SHIP-1 activity. This is indicated by the absence of SHIP-1 phosphorylation at tyrosine residue 1020 as well as by the activation of PI3K pro-survival pathways in TB compared to control splenocytes. Results in our model are consistent with findings that suggest that the absence of SHIP-1 expression results in perturbed cytokine levels that influence and regulate the outcome of immune responses [41].

IL-6 and MCP-1 have been characterized as key soluble factors in MDSC induction and migration, respectively [42], [43]. SHIP deficient mice have an increase in IL-6 production in their serum, which skews hematopoiesis, causes a suppression of B lymphopoiesis and increases cytokine-induced myeloid proliferation [7], [21], [44]. SHIP-1 has also been found to negatively regulate MCP-1 production *in vitro* [45]. In our TB mice, we report a significant increase in both IL-6 and MCP-1 in their serum. We also report an expansion of MDSC in the peripheral blood and splenocytes of TB mice, but a reduction in their B cell percentages (*data not shown*). It therefore appears that our TB mice show multiple defects in myelopoiesis and lymphopoiesis that may be due to a reduction in SHIP-1 expression similar to that observed in SHIP deficient mice [20].

Expanded myeloid cells are more resistant to apoptosis in SHIP deficient mice [15]. In our study, the hyper-phosphorylation of AKT-1 and over expression of BCL-2 in sorted TB MDSC (Gr-1^+^ cells) suggests that these cells may be resistant to programmed cell death causing them to accumulate in the spleen. It also appears that these TB MDSC are functionally more suppressive compared to control, on a cell-to-cell basis. This indicates a possible correlation between SHIP-1 expression and loss of MDSC homeostasis in this model.

Ghansah et al reported a loss of homeostasis and function of MDSC in SHIP deficient mice whereas Collazo et al detailed similar findings in Treg [16], [19]. Huang et al reported that a sub-population of MDSC causes the induction and expansion of immunosuppressive Treg that also suppress T cell immune responses [46]. Treg play a key role in maintenance of self-tolerance and immune homeostasis [47]. It is possible that release of IL-10 and other unidentified soluble factors from murine Panc02 cells may lead to the induction and activation of Treg in TB mice. We know based on flow cytometry studies that there is a significant increase in CD4^+^CD25^+^CD127^−^ Treg population in PB and splenocytes (*data not shown*) of TB mice as compared to control mice. We are further characterizing these Treg by evaluating their expression of the transcription factor Foxp3 and their ability to suppress antigen-specific T cell responses.

These findings give insight into the regulation of MDSC in a murine pancreatic cancer microenvironment. Our findings also highlight that there is a possible role of SHIP-1 in solid tumor progression and as a tumor suppressor in this pancreatic cancer model. This may translate to other cancer models in which this particular phenotype is observed. Tumor-induced MDSC have been a challenge to many immunotherapeutic strategies including cancer vaccines. Our study opens a new therapeutic avenue of potentially targeting SHIP-1 expression, which may directly or indirectly alleviate immune suppression associated with MDSC. Therefore, the use of therapeutic agents that modulate SHIP-1 expression will provide more insight into the possible role of SHIP-1 as a tumor suppressor in solid tumors such as pancreatic cancer.

## Materials and Methods

### Cell culture

The Panc02 murine pancreatic adenocarcinoma cell line was established by Corbett et al. by implanting cotton thread impregnated with 3-methylcholanthrene into the pancreas of C57BL/6 mice, followed by serial *in vivo* passage through repeated transplantation [48]. This cell line was maintained in RPMI 1640 medium supplemented with 10% fetal bovine serum (FBS), (HyClone, Logan, UT, USA), 2 mM L-glutamine, 100 U/ml penicillin, 100 µg/ml streptomycin (Gibco BRL, Rockville, MD, USA) at 37°C in 5%CO_2_. Cultured cells were tested and found to be negative for mycoplasma and viral contamination.

### Mice

Pathogen-free female C57BL/6 (6–8 weeks old) mice were purchased from Harlan Laboratories, Inc (Indianapolis, IN). OT-I mice were purchased from The Jackson Laboratory (Bar Harbor, ME). All mice were maintained in a pathogen-free animal facility for at least 1 week before each experiment. The Institutional Animal Care and Use Committee of the University of South Florida approved all protocols in compliance with the Guide for the Care and Use of Laboratory Animals. C57BL/6 mice were injected subcutaneously (s.c.) with 1.5×10^5^ Panc02 murine cells (tumor-bearing (TB)) or 100 µl PBS (control) on the right ventral abdomen. Established tumors were measured every three days using a digital caliper and spleens were harvested and weighed at the end of the study.

### Cytometric Bead Array Immunoassay

Peripheral blood was collected from control and TB mice and allowed to clot at room temperature for 2 hours. Serum was centrifuged at 2,000 rpm for 10 minutes, collected and stored at −20°C until assays were performed. Supernatant was collected from cultured Panc02 cells. Serum and supernatant concentrations of IL-6, IL-10, MCP-1, IFN-γ, TNF and IL-12p70 were simultaneously measured using a CBA Mouse Inflammation Kit (BD Biosciences, San Diego, CA). Briefly, 50 µL of chemokine capture bead mixture was incubated with 50 µL of each recombinant standard or sample and 50 µL PE-conjugated detection antibody for 2 hours at room temperature. The mixture was then washed to remove unbound PE detection reagent before data acquisition on a BD LSRII using flow cytometry. Analysis was performed using FCAP Array software.

### Western Blot Analyses

Splenocytes were harvested from control and TB mice and single-cell suspensions were prepared using a Cell Dissociation Sieve (Tissue Grinder) (Sigma-Aldrich) and 70 µm cell strainers (BD Falcon). Red Blood Cells (RBCs) were lysed using RBC Lysis Buffer (eBioscience). Whole and Gr-1^+^ (MDSC) enriched splenocytes from control and TB mice were made into protein lysates using modified RIPA Buffer (Millipore, MA) containing Na_3_OV_4_ and protease inhibitor cocktail (Sigma-Aldrich, MO). Protein concentrations of these lysates were determined using the BCA Protein Assay Kit (Thermo Fisher Scientific, IL), according to the manufacturer's protocol. 15 µg and 60 µg of whole and Gr-1^+^ enriched protein lysates, respectively, were loaded per lane, resolved using NuPAGE® 4–12% Bis-Tris polyacrylamide Gels (Invitrogen, CA) and transferred to a nitrocellulose membrane (Whatman, NJ). The membranes were blocked with 5% nonfat milk, 1XPBS and 0.1% Tween-20 and then probed with the following antibodies: anti-SHIP-1 (PIC1) (Santa Cruz, CA) at a 1∶200 dilution, anti-Phospho-AKT-1 (Ser473) (Santa Cruz, CA) at a 1∶200 dilution, anti-AKT1/2/3 (Santa Cruz, CA) at a dilution of 1∶200, anti-Phospho BAD (Ser112) (Santa Cruz, CA) at a dilution of 1∶200, anti-BAD (Santa Cruz, CA) at a dilution of 1∶200, anti-BCL-2 at a dilution of 1∶1000 (Cell Signaling, MA), anti-SHIP-2 at a dilution of 1∶1000 (Cell Signaling, MA), anti-PTEN at a dilution of 1∶1000 (Cell Signaling, MA) Proteins were detected by secondary antibodies, anti-rabbit IgG, anti-mouse IgG HRP-linked, (Cell Signaling, MA), at a dilution of 1∶1000 or anti-goat IgG HRP-linked at a dilution of 1∶5000 (Santa Cruz, CA). Secondary antibodies were identified using Super Signal West Pico and Femto Chemiluminescent Substrate (Thermo Fisher Scientific, IL), according to the manufacturer's protocol. All blots were stripped and re-probed with anti-β-actin (Sigma-Aldrich, MO) at a dilution of 1∶20,000, as an internal control for equal protein loading. Membranes were exposed to x-ray films (Phenix, NC) and developed using a Kodak M35-X OMAT Processor. Western blot bands were quantified using a ChemiDoc XRS Imaging System and Quantity One 1-D densitometry analysis software (Bio-Rad, CA).

### Quantitative RT-PCR

Single-cell suspensions of whole splenocytes from control and TB mice were prepared as previously described. Total RNA was extracted using TRI Reagent (Molecular Research Center), according to the manufacturer's protocol. cDNA was synthesized using the High Capacity cDNA Reverse Transcription Kit (Applied Biosystems) from total RNA (1 µg). mRNA levels for SHIP-1 were detected by qRT-PCR using SYBR Green JumpStart *Taq* Ready Mix (Sigma-Aldrich) and an AB StepOnePlus Real-Time PCR System using the following conditions: 95°C for 10 min followed by 40 cycles of 95°C for 15 sec and 60°C for 1 min. The following primers were used: SHIP-1 forward, 5′-GAG CGG GAT GAA TCC AGT GG-3′, reverse, 5′-GGA CCT CGG TTG GCA ATG TA-3′. Each sample was assayed in triplicate. GAPDH was amplified as the internal control and reference gene. The relative mRNA frequency was determined by normalization to the endogenous control GAPDH and calculated using the Comparative C_T_ method.

### *In vitro* Panc02 Experiments

Single-cell suspensions of control splenocytes were prepared as previously described. 5×10^5^ control splenocytes were co-cultured *in vitro* with confluent murine Panc02 cells or cultured alone in 6-well plates for 5 hours. Splenocytes in suspension were separated from adherent Panc02 cells and analyzed for SHIP-1 mRNA and protein expression using qRT-PCR and Western Blot analyses, as previously described.

### Flow Cytometry

Single-cell suspensions of whole splenocytes from Control and TB mice were prepared as previously described and were stained in PBS/3%FBS with antibodies against MDSC surface markers CD11b (Mac-1) (FITC) (eBioscience), Ly6G and Ly6C (Gr-1) (APC) (BD Pharmingen). Flow Cytometry was done using a BD LSRII Flow Cytometry System (BD Biosciences Immunocytometry Systems) and data analyzed with FlowJo software (Tree Star Inc.)

### AutoMACS Gr1^+^ Enrichment and Suppression Assay

Whole splenocytes from control and TB mice were processed into single-cell suspensions, as previously described. Gr-1^+^ cells were purified from whole splenocytes by positive selection using anti-Gr-1-PE and anti-PE-magnetic microbeads on an AutoMACS Pro Separator. Sorted control and TB MDSC were co-incubated 1∶10 with OT-I specific (CD8^+^) T cells primed with bone marrow-derived dendritic cells pulsed with 10 µg/ml OVA_SIINFEKL_ peptide (DC-OVA), for 3 days *in vitro*. T cell proliferation was measured by ^3^H incorporation during the final 12 hours of the culture. Controls for the assay included OT-I specific T cells incubated alone in complete medium (CM) or with DC pulsed with OVA peptide (DC-OVA) [49].

### Statistical Analysis

All *in vitro* experiments described in this study are representative of at least three independent analyses. All *in vitro* assays and flow cytometry results were analyzed with two-tailed Student's *t* test using PRISM 5 software (GraphPad, San Diego, CA). Differences were considered significant at p<0.05.
